# Three-Month History of Lymphadenopathy Caused by Bartonella henselae in a 13-Year-Old Following a Dog Scratch

**DOI:** 10.7759/cureus.66134

**Published:** 2024-08-04

**Authors:** Martin Nguyen, Sheraj Singh, Bevan Sam, Richard Llerena, Abigail Frank, Marinella Mabalot

**Affiliations:** 1 Radiology, West Virginia School of Osteopathic Medicine, Lewisburg, USA; 2 Medicine, West Virginia School of Osteopathic Medicine, Lewisburg, USA; 3 Clinical Sciences, West Virginia School of Osteopathic Medicine, Lewisburg, USA; 4 Family Medicine, West Virginia School of Osteopathic Medicine, Lewisburg, USA; 5 Family Medicine, Greenbrier Valley Medical Center, Ronceverte, USA

**Keywords:** differential for fever of unknown origin, head and neck mass, bartonella, supraclavicular lymphadenopathy, cat-scratch disease

## Abstract

We reported the case of a 13-year-old immunocompetent boy presenting with a right cervical neck mass. He complained of fatigue, back pain, coughing, and a right neck mass persisting for three months. He did not have a fever, but his parents reported he had lost 20 lbs. in the past six months without any change in diet or appetite. They are also very concerned about the risk of malignancy. During the initial work-up, there was no abnormality in the complete blood count. During the follow-up visit 10 days later, he complained of new-onset dysphagia and throat pain. The mass was about 5 cm on the right neck, poorly mobile, and mildly tender to palpation. It looks significantly different compared to the first visit. Blood serology tests were indicated, and titers of cytomegalovirus (CMV), Epstein-Barr virus (EBV), and toxoplasma were not reactive. However, serology detected that IgM and IgG titers to *Bartonella henselae* were ≥1:20 and ≥1:1024, respectively. A fine needle aspiration (FNA) of the mass on the same day revealed lymphoid proliferation. Afterward, the patient was treated with amoxicillin-clavulanic acid for two weeks. After three weeks, the mass almost disappeared, and the patient reported a remarkable improvement in symptoms. This case report is a helpful reminder that *B. henselae* should be suspected on the differential diagnoses in a case of lymphadenopathy associated with non-specific symptoms such as fatigue, back pain, and weight loss.

## Introduction

Bartonellosis is a group of infectious diseases caused by the genus *Bartonella* with at least six species that may cause human disease [1]. These microorganisms are intracellular, Gram-negative, and oxidase-negative bacilli [1]. One of the most common causes of cat-scratch disease (CSD) is *Bartonella henselae*. This bacterium was described about 30 years ago and may cause bacillary angiomatosis (BA), endocarditis, and CSD [2]. During the past three decades, several cases of isolation of *Bartonella *species from blood and lymph in patients with CSD have been reported with confirmation by indirect fluorescent antibody (IFA), enzyme-linked immunosorbent assay (ELISA), or polymerase chain reaction (PCR) [3]. *B. henselae* is also associated with many other clinical manifestations [1,4]. It is transferred between cats by the cat flea, *Ctenocephalides felis* [5]. CSD is often a self-limiting and asymptomatic disease. Sometimes, it may present with flu-like symptoms with or without regional lymphadenopathy [1]. Treatment is mainly supportive, and antibiotics may be indicated in severe disease or those with systemic manifestations [1,4].

## Case presentation

**Figure 1:**
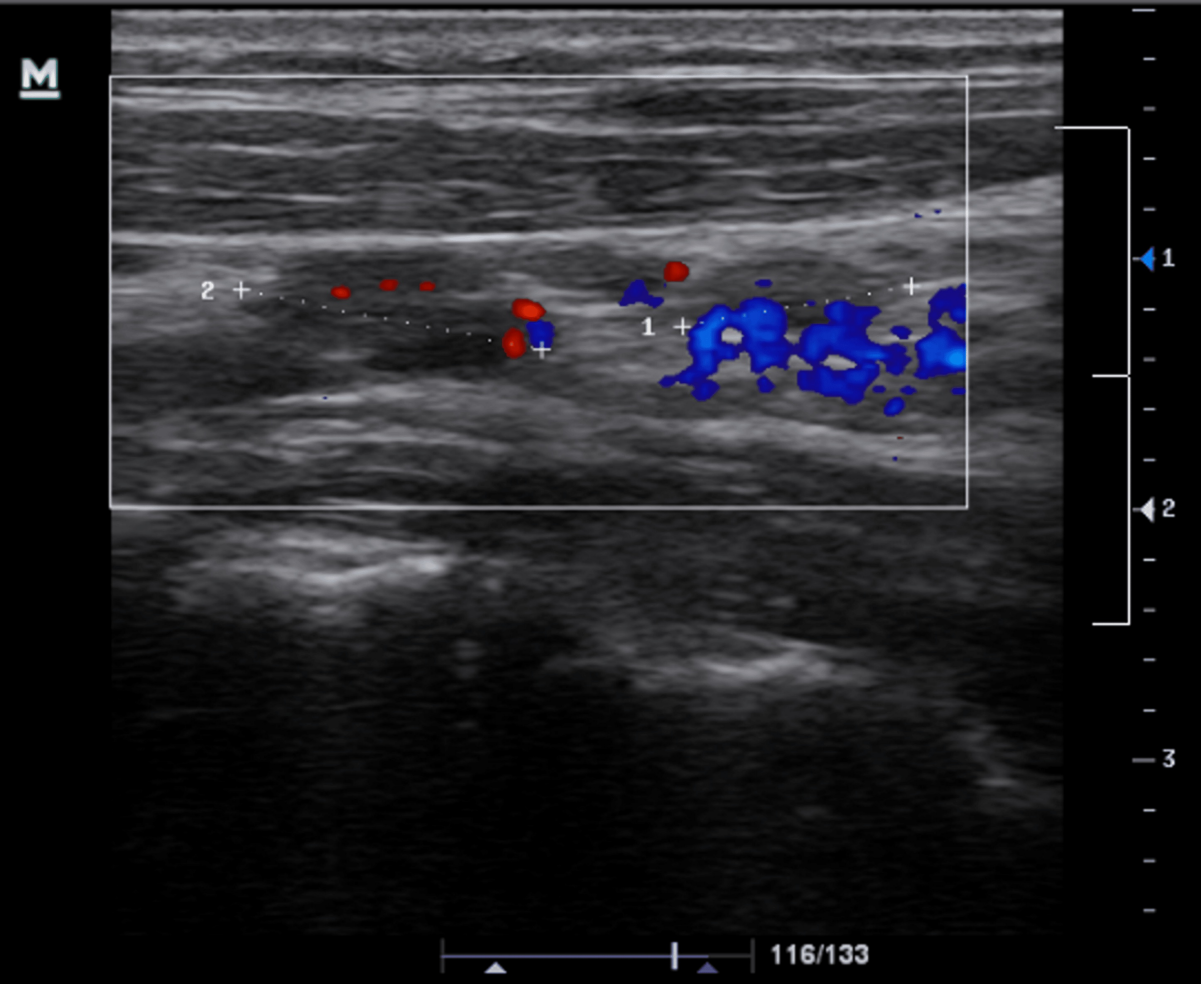
Bilateral lymphadenopathy was demonstrated on ultrasound on the initial visit.

A 13-year-old immunocompetent male presented to our clinic with a chief complaint of a neck mass on the right. He was accompanied by his parents. He also had a one-day dry cough with nasal congestion. Regarding the neck mass, he noticed that it had been there for three months and had become bigger recently. He denied any fever, chills, fatigue, or night sweats. His mother stated that he lost 20 lbs. in six months without any change in diet or appetite. She is concerned about him acquiring some form of childhood malignancy. When being questioned about any possible cat exposure, he stated that a dog scratched him a few months ago. The patient did not have any autoimmune diseases or a history of recurrent infections. His travel history was unremarkable. On examination, his vital signs were normal. Height and weight were 67th and 83rd percentile, respectively. A 3-cm neck mass, mobile, was detected on the right medial clavicle. No axillary adenopathy was detected. Physical examination was unremarkable. Complete blood count was unremarkable (Table 1). Ultrasound was indicated and demonstrated bilateral lymphadenopathy (Figure 1). The largest lymph nodes (LNs) in the left and right supraclavicular regions were 2.4 cm and 2.3 cm, respectively. He was suspected of possible CSD and was indicated azithromycin for five days (500 mg on the first day and 250 mg/day from days 2 to 5).

**Table 1:** Complete blood count at the initial visit. WBC: white blood cell; RBC: red blood cell; Hb: hemoglobin; Hct: hematocrit; MCV: mean corpuscular volume; MCH: mean corpuscular hemoglobin; MCHC: mean corpuscular hemoglobin concentration; RDW: red cell distribution width; PLT: platelet; NEU: neutrophil; LYMPH: lymphocyte; MONO: monocyte; EOS: eosinophil; BASO: basophil

Parameters	Results	Reference range	Units
WBC	3.8	3.8-10.7	K/mm^3^
RBC	4.55	3.9-5.8	M/mm^3^
Hb	12.3	12.0-17.5	g/dL
Hct	37.4	35.8-52.9	%
MCV	82.3	81-99	fL
MCH	27.1	26-34	pg
MCHC	32.9	32-35	g/dL
RDW	16.6	12.6-15.7	12.6-15.7
PLT	285	150-450	K/mm^3^
NEU # (NEU %)	1.8 (46.6)	1.9-7.3 (40.8-77.3)	K/mm^3^ (%)
LYMPH # (LYMPH %)	1.4 (38.2)	0.9-3.8 (14.5-48.7)	K/mm^3^ (%)
MONO # (MONO %)	0.5 (13.6)	0.2-0.9 (4.0-11.9)	K/mm^3^ (%)
EOS # (EOS %)	0.02 (0.5)	0.0-0.5 (0.4-7.3)	K/mm^3^ (%)
BASO # (BASO %)	0.0	0.0-0.1 (0.0-1.6)	K/mm^3^ (%)

**Figure 2:**
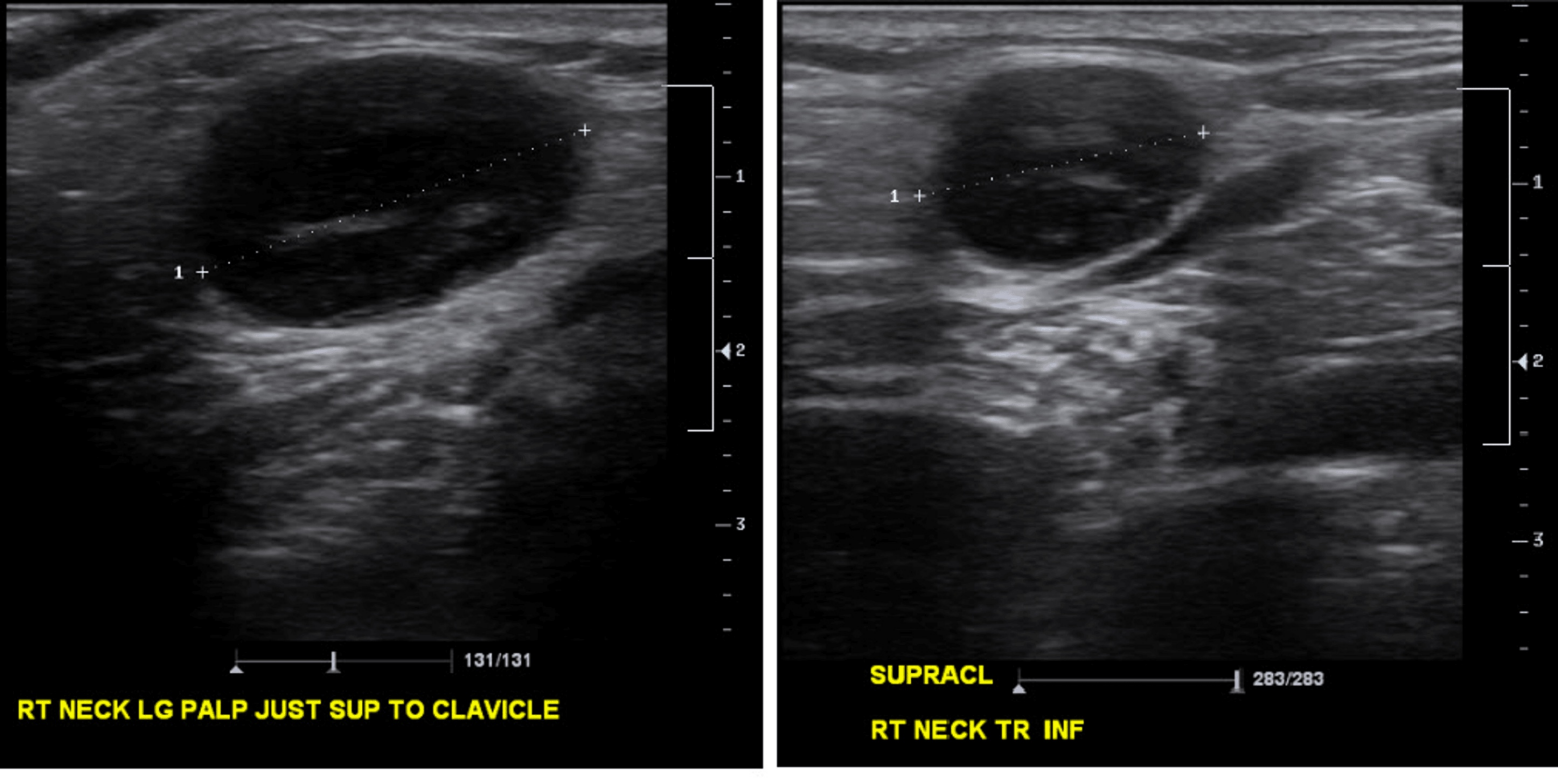
(Left) Right supraclavicular lymph node before treatment (largest diameter about 2.3 cm). (Right) Right supraclavicular lymph node two weeks after treatment initiation (largest diameter about 1.6 cm).

Ten days later, at the subsequent visit, the neck mass had increased significantly. The patient also complained of new-onset dysphagia and throat pain. It was about 5 cm in largest diameter, poorly mobile, and mildly tender to palpation. Fine needle aspiration (FNA) was indicated under ultrasound guidance. The result was lymphoid proliferation without any findings suggesting an underlying malignant pathology. Serology results showed that antibodies against *B. henselae *were increased (IgM ≥1:20, IgG ≥1:1024). Additionally, titers for cytomegalovirus (CMV), toxoplasma, and Epstein-Barr virus (EBV) were negative. Amoxicillin-clavulanic acid was added to the regimen (875 mg/125 mg, twice a day) for two weeks. Another five-day course of azithromycin was indicated. In the follow-up visit two weeks later, the mass was noted to decrease significantly in size (2.0×1.5 cm on clinical examination). The rest of the head and neck exam was unremarkable. A repeat ultrasound demonstrated the largest LNs in the left and right supraclavicular regions were 1.1 cm and 1.6 cm, respectively. Other LNs ranged from 0.6 cm to 1.1 cm on both sides of the neck. These LNs had significantly decreased in size compared to the previous study (Figure 2).

## Discussion

Typical CSD refers to a syndrome characterized by isolated lymphadenopathy with fever and no other signs or symptoms [1]. After 3-5 days of exposure to a cat, erythematous papules may occur at the inoculation site. Regional lymphadenopathy occurs 1-3 weeks after inoculation [1,6,7]. Entrance may be acquired through a wound, a skin break, or a scratch or bite of a cat or other animals [7]. In the majority of cases, CSD often involves a single LN, especially axillary and epitrochlear LNs (46%), head and neck LNs (26%), and groin LNs (17.5%) [7]. The nodal distribution may be explained by the fact that cat contact most often occurs with the hands and upper limbs [1]. Our patient had a 5-cm, poorly mobile, supraclavicular neck mass on the right. There were no signs of suppuration noted. In a sample of 1,200 CSD patients, Carithers [7] reported fever is present in 59% of all cases and <10% experiencing fever exceeding 39°C. Lymphadenopathy was present in all the patients, 85% of whom presented with a single LN involvement. A history of cat exposure was reported to be 99.1%, and an inoculation site was detected in 92.6% of the cases [7]. Suppuration was also noted in 10-11.8% of cases, and drainage is required in those cases [7]. In more than 75% of CSD cases, the presentation was mild with aching, malaise, and anorexia and rarely associated with nausea and abdominal pain [7].

CSD is associated with systemic symptoms in 5-10% of all cases and may lead to complications such as fever of unknown origin (FUO), hepatosplenic involvement, encephalopathy, osteomyelitis, and ocular diseases [1,8]. *B. henselae *is one of the most important causes of FUO [9]. FUO is defined as a fever lasting for >2 weeks without any signs or symptoms of an obvious clinical disease [1]. In a prospective study of 146 children spanning over six years (1990-1996), Jacobs and Schutze [9] reported that the three most common causes of infection causes included EBV infection (15.1%), osteomyelitis (9.6%), and bartonellosis (4.8%). Approximately 30% of FUO cases caused by *B. henselae *had hepatosplenic complications [1]. Thus, in cases of FUO and abdominal pain, *B. henselae *infection should always be considered in the differential diagnoses, especially in those with previous cat exposure [1,9]. In some rare instances, symptoms of meningoencephalitis, endocarditis, and ocular complications have been reported in immunocompromised patients [10-12]. Encephalopathy due to CSD may present as severe headache and acute confusion with or without seizures. These episodes often occur 1-6 weeks after the appearance of lymphadenopathy [12]. The most common ocular involvement is Parinaud oculoglandular syndrome, which comprises conjunctivitis and ipsilateral periauricular lymphadenopathy [11]. Acute visual loss may occur due to optic nerve edema and macular exudates [11]. Two other clinical entities of CSD in immunocompromised patients include BA and bacillary peliosis (BP). BA involves skin and LN lesions while BP affects the liver and sometimes the spleen [5]. BA was first described in patients with acquired immunodeficiency syndrome (AIDS) with very low CD4 cell counts [13]. This population's most common *Bartonella *species include *B. quintana *and *B. henselae *[13].

Radiologic manifestations of CSD may include lymphadenopathy, hepatosplenic lesions, osteomyelitis, neuroretinitis, cranial nerve neuritis, encephalitis, and meningitis [14]. On ultrasound of typical CSD, LNs often appear as hypoechoic structures which are highly vascularized with increased echogenicity of the surrounding soft tissue [14]. In this case, ultrasound demonstrated bilateral lymphadenitis with multiple LNs of varying size. The largest LN could be palpated as a right cervical neck mass (5 cm) near the medial clavicle. Biopsy of LNs in a case of typical CSD may demonstrate granuloma appearance with multiple microabscesses [1]. Moreover, abdominal imaging is an important diagnostic step in suspected cases of hepatosplenic disease or prolonged FUO [1]. Abdominal ultrasound and computed tomography (CT) may detect liver and/or spleen microabscesses in more than 50% of patients with hepatosplenic CSD [1,15]. Abdominal imaging was not indicated in our case because there were no suggestive symptoms or signs, e.g., fever or abdominal pain.

Because of a wide range of clinical manifestations and a lack of a diagnostic gold standard, it is challenging to diagnose CSD [6]. Diagnosis generally relies on a detailed history of exposure, clinical findings, serology, histology, and imaging criteria. Our patient had a documented history of a dog scratch and positive serology against *B. henselae *(IgM ≥1:20 and IgG ≥1:1024). An aspiration of the neck mass demonstrated lymphoid proliferation without any suggestive findings of an underlying malignant pathology. Additionally, the tuberculin skin test (TST) was negative. In 2000, Margileth [16] proposed a criteria system for CSD diagnosis. Our patient satisfied three criteria (history of dog scratch, negative TST, and positive serology against *B. henselae*) (Table 2). It is important to note that dogs have been demonstrated as reservoirs for *B. henselae*. In 1998, Tsukahara et al. [17] reported a case of *B. henselae* infection from a dog being confirmed by PCR. Hence, the name "cat-scratch disease" may lead to a misunderstanding that *B. henselae *is only present in cats. Regarding the differential diagnoses, some common etiologies of unilateral lymphadenopathy are listed in Table 3. In our case, another notable finding is the weight loss, which may be suggestive of a malignant process. There are several reports in the literature that CSD may mimic various malignancies, especially when the patients present with lymphadenopathy in the neck and abdomen [18-20]. The most confusing clinical picture is when the patients have a constellation of symptoms called "B symptoms" of lymphoma which may include weight loss, night sweats, and prolonged fever [21]. Besides, Hodgkin lymphoma often presents with painless regional lymphadenopathy. Our patient had 20 lbs. weight loss in six months, but he denied any night sweats or fever. Complete blood count was in the normal range. Furthermore, the FNA result yielded lymphoid proliferation without any findings suggesting an underlying malignancy. Initially, the patient was indicated for an excisional biopsy (EB). However, after a detailed history, combined with consistent results of serology, ultrasound, and FNA, EB was deemed unnecessary. In terms of radiology, CSD shares many similar features with other pathologies such as lymphoma and tuberculosis. However, when combined with history and pathology, radiology will be very helpful in differential diagnoses, especially in the hands of experienced radiologists [20].

**Table 2:** Criteria for the diagnosis of CSD. CSD: cat-scratch disease; CT: computed tomography; IFA: indirect fluorescent antibody; PCR: polymerase chain reaction Source: Margileth [16]

Three of four of the following:
Cat or flea contact with/without a scratch mark or a regional inoculation lesion (skin papule, eye granuloma, mucous membrane)
Laboratory/radiology: negative purified protein derivative or serology for other infectious causes of adenopathy; sterile pus aspirated from a node, PCR assay positive; *Bartonella henselae*, *Bartonella quintana*, or *Afipia felis*: highest sensitivity. CT scan: liver/spleen abscesses
A positive enzyme immunoassay or IFA assay serology test >1:64 for *Bartonella henselae*, *Bartonella quintana, *or *Bartonella clarridgeiae*; a fourfold rise in titer between acute and convalescent specimens is definitive
Biopsy of node, skin, liver, bone, or eye granuloma showing granulomatous inflammation compatible with CSD; positive Warthin-Starry silver stain

**Table 3:** Common diseases that may cause unilateral lymphadenopathy. CMV: cytomegalovirus; EBV: Epstein-Barr virus; HIV: human immunodeficiency virus Source: Klotz et al. [5]

Common diseases that may cause unilateral lymphadenopathy
Infectious causes
CMV lymphadenopathy
EBV lymphadenopathy
Group A streptococcal adenitis
HIV lymphadenopathy
Nontuberculous mycobacterial lymphadenitis
*Staphylococcus aureus* adenitis
Toxoplasmosis lymphadenopathy
Noninfectious causes
Malignancy (lymphoma, leukemia)

There is not enough data regarding the best therapeutic regime in all cases of *B. henselae*. Most data was gleaned from case series rather than randomized controlled trials (RCTs). Additionally, there is also a big gap between in vitro and clinical success. For example, penicillin has a very low mean inhibitory concentration (MIC) in vitro but demonstrated no clinical efficacy [1]. Typical CSD is a self-limited disease that tends to resolve within 2-6 months. Most studies reported that antibiotics showed no significant benefit [1]. However, at least two studies revealed some clinical effectiveness. In a retrospective analysis of 268 patients, Margileth [22] reported four effective antibiotics to treat CSD with noticeable clinical efficiency as follows: rifampin (87%), ciprofloxacin (84%), gentamicin (73%), and trimethoprim-sulfamethoxazole (58%). The mean period of adenopathy in those who were not treated with antibiotics was 14.5 weeks, and the mean duration of those treated with one of the four antibiotics above was 2.8 weeks [22]. The effectiveness of antibiotic therapy was based on clinical improvement which included a decrease or absence of malaise, fever, fatigue, headache, anorexia, lymphadenopathy, and an improvement in sedimentation rate. The author suggested antibiotics may be indicated in severe cases of CSD, while mild to moderate cases should be managed with symptomatic treatment and regular follow-ups [22]. In an RCT to investigate the efficacy of azithromycin in CSD, patients with typical CSD were randomized to be treated with oral azithromycin for five days (group 1) or placebo (group 2). There was a decrease in LN volume in 50% of the cases in group 1 compared to 7% in group 2 (p=0.026) in the first 30 days [23]. After 30 days, there were no differences regarding the outcome between the two groups [23]. In our patient, he was treated with amoxicillin-clavulanic acid (875 mg/125 mg, twice a day) for two weeks and azithromycin for five days (500 mg the first day and 250 mg/day from days 2 to 5).

In most cases of CSD, the prognosis is excellent with spontaneous clinical recovery within several months. Lymphadenopathy often resolves within 2-6 months, but sometimes it may take 1-2 years [22]. One episode of CSD tends to confer lifelong immunity in children and adolescents. However, recurrence of CSD has been reported [24]. We reassured the patient and his parents regarding his prognosis. Meanwhile, we must be aware of the possibility of the persistence of *Bartonella *species in humans. In 2017, Velho et al. [2] reported the case of a 10-year-old girl with a 3-cm neck mass on the right. History and serology findings suggested she had CSD, and she was promptly treated with doxycycline for six weeks. Clinical improvement was noted afterward. However, a new left mass occurred in the left cervical region four years later. CT evaluation demonstrated multiple cervical lymphadenopathy, mesenteric lymphadenopathy, and splenomegaly. *B. henselae *(strain Brazil-1) DNA was confirmed by real-time PCR. She was diagnosed with chronic lymphadenopathy due to bartonellosis. After being discharged, she was treated with azithromycin (500 mg/day) for one year. However, bilateral cervical enlargement remained till she was at least 20 years old, and she was still being followed up regularly at that time. This case underscored a rare chance of long-term persistence of *B. henselae *in humans.

## Conclusions

Over the last few decades, CSD has been diagnosed in clinical settings more frequently. It is among the most common causes of infectious lymphadenitis in children and adolescents. In general, CSD is a self-limiting illness with an excellent prognosis in the majority of cases. Early diagnosis and treatment may help reduce both morbidity and mortality of potentially rare CSD complications. The key to diagnosis still relies mainly on a detailed history combined with appropriate clinical, histopathologic, serologic, and radiologic findings. We presented this case to serve as a reminder of a few notable points: (1) CSD is a common cause of lymphadenopathy in children and adolescents, (2) CSD may mimic many other diseases, especially several childhood malignancies, and (3) the best antibiotic approach for severe cases is still a matter of debate with no clear or useful guidelines. The final take-home point is that CSD should always be considered in a child presenting with FUO and abdominal pain.
